# Penetration of Isavuconazole in Ascites Fluid of Critically Ill Patients

**DOI:** 10.3390/jof7050376

**Published:** 2021-05-11

**Authors:** Tobias Lahmer, Gonzalo Batres Baires, Roland M. Schmid, Johannes R. Wiessner, Jörg Ulrich, Maximilian Reichert, Wolfgang Huber, Fritz Sörgel, Martina Kinzig, Sebastian Rasch, Ulrich Mayr

**Affiliations:** 1 Klinik und Poliklinik für Innere Medizin II, Klinikum rechts der Isar der Technischen Universität München, Ismaningerstrasse 22, 81675 Munich, Germany; Gonzalo.Batres@mri.tum.de (G.B.B.); Roland.Schmid@mri.tum.de (R.M.S.); Johannes.wiessner@mri.tum.de (J.R.W.); joerg.ulrich@mri.tum.de (J.U.); maximilian.reichert@mri.tum.de (M.R.); wolfgang.huber@mri.tum.de (W.H.); sebastian.rasch@mri.tum.de (S.R.); Ulrich.Mayr@mri.tum.de (U.M.); 2 IBMP—Institute for Biomedical and Pharmaceutical Research, Paul-Ehrlich-Straße 19, 90562 Nürnberg-Heroldsberg, Germany; Fritz.Soergel@ibmp.net (F.S.); martina.kinzig@ibmp.net (M.K.); 3 Faculty of Medicine, Institute of Pharmacology, University Duisburg-Essen, Hufelandstrasse 55, 45122 Essen, Germany

**Keywords:** isavuconazole, peritonitis, intra-abdominal infection, fungal peritonitis, ascites

## Abstract

Fungal peritonitis is a life-threatening condition which is not only difficult to diagnose, but also to treat. Following recent guidelines, echinocandins and azoles are the recommended antimycotics for the management of intra-abdominal *Candida* spp. infections, with a favor for echinocandins in critically ill patients. However, the new extended spectrum triazole isavuconazole also has a broad spectrum against *Candida* spp. Data on its target-site penetration are sparse. Therefore, we assessed isavuconazole concentrations and penetration ratios in ascites fluid of critically ill patients. Obtaining of Isavuconazole plasma and ascites fluid levels as well penetration ratios using paracentesis in critically ill patients. Isavuconazole concentrations were quantified in human plasma and ascites by a liquid chromatography/tandem mass spectrometry (LC-MS/MS) method. Isavuconazole concentrations in plasma and ascites fluid were measured in sixteen critically ill patients. Isavuconazol levels in ascites fluid (1.06 µg/mL) were lower than plasma levels (3.08 µg/mL). Penetration ratio was 36%. In two out of sixteen patients, *Candida* spp., in detail C. glabrata and C. tropicalis, could be isolated. Cmax/MIC Ratio in plasma of 560 for C. glabrata and 2166 for C. tropicalis could be observed. Following our results, isavuconazole penetrates into ascites. Successful treatment in *Candida* spp. peritonitis depends on pathogen susceptibility.

## 1. Introduction

Intra-abdominal candidiasis (IAC) or following fungal peritonitis is poorly understood compared with other fungal infections such as candidemia. Until now, data and studies on the efficacy of treatment options in IAC are scarce and recommendations in current international guidelines mainly address the findings for candidemia as equal for IAC.

In fact, fungal peritonitis is associated with an overall relevant increase in morbidity and mortality [1]. Beyond this, it is associated with tremendous higher mortality rates in special patient groups, e.g., patients with end stage liver disease [2]

Following recent guidelines, echinocandins and azoles are the recommended antimycotics for the management of intra-abdominal *Candida* spp. infections, with a favor for echinocandins in critically ill patients [3]. Thus, the new extended spectrum triazole isavuconazole has, beside its approval for the treatment of invasive aspergillosis and mucormycosis, also a broad spectrum against *Candida* spp. [4,5].

Although, there are some data of tissue penetration into tissue (e.g., brain and lungs), these data are mostly based on animal experiments [6,7].

However, data assessing tissue penetration of antifungals at infected tissue sites in humans, especially into ascites fluid, are lacking in general, and of isavuconazole in detail. 

The aim of this observational study within critically ill patients is to investigate the penetration ratios of isavuconazole into ascites fluid (I) and the effectiveness in case of *Candida* spp. peritonitis (II). 

## 2. Materials and Methods

Between February 2019 and November 2019, paracentesis was performed in sixteen severe neutropenic critically ill patients (age 60 ± 15 years; male gender *n* = 11) with underlying hematological malignancies. The main reason for ICU admission was septic shock caused by pneumonia (APACHE II score 22 ± 5; SOFA Score 11 ± 3) with need for mechanical ventilation, broad spectrum antibiotics and catecholamines in all patients. Seven out of 16 patients needed renal replacement therapy during ICU stay. This leads to a mean ICU stay of 20 ± 8 days and a mortality rate of 88%.

This study on critically ill adults with an indication for isavuconazole treatment was approved by the institutional review board of the Technical University of Munich, Germany (87/20S). Written informed consent was obtained by the patient or their legal representatives.

Based on the critical illness combined with temporarily gastroesophageal reflux/mucositis complications, isavuconazole was applicated through central venous catheters as recommended by the manufacturer (For day 1 and 2, 200 mg every eight hours, then 200 mg per day). Blood samples were taken from an arterial line using EDTA vials, then were centrifugated and stored with the ascites samples at −70 °C.

Isavuconazole concentrations were quantified in human plasma and ascites by a liquid chromatography/tandem mass spectrometry (LC-MS/MS) method using an API 5000 triple quadrupole mass spectrometer (SCIEX, Concord, Ontario, Canada). Fifty µL of each sample was deproteinized with 300 µL acetonitrile (containing the internal standard isavuconazol-d4), and subsequently vortex-shaked and centrifuged. The supernatant was further diluted with Milli-Q^®^ water (1:190, *v*/*v*), and 10 µL of each samples were injected into the LC-MS/MS system.

During validation, the linearity for isavuconazole in human plasma could be demonstrated over a calibration range from 0.107 to 9.68 µg/mL. The inter-day precision for isavuconazole SQCs in human plasma was 2.2% at 8.89 µg/mL, 3.5% at 4.45 µg/mL, 5.4% at 0.737 µg/mL, and 7.6% at 0.147 µg/mL. The inter-day accuracy in human plasma ranged from 94.1 to 106.0%. The intra-day-precision of SQC 1 to SQC 4 ranged from 2.3 to 5.7%, and the intra-day accuracy from 93.7.1 to 99.4%. No interference at the retention time of isavuconazole and the internal standard could be observed in drug free human plasma samples from critical ill patients.

The linearity for isavuconazole in human ascites could be demonstrated over a calibration range from 0.194 to 10.50 µg/mL. Since all samples for ascites were analyzed on one day only an intra-day precision was performed. The intra-day precision for isavuconazole SQCs in human ascites was 3.5% at 10.1 µg/mL, 30.9% at 2.07 µg/mL and 6.6% at 0.696 µg/mL. The intra-day accuracy in human ascites ranged from 100.0 to 103.3%. No interference at the retention time of isavuconazole and the internal standard could be observed in drug free human ascites samples from critical ill patients.

For the single samples obtained at paracentesis, the ratio between the concentration in ascites fluid and the plasma level concentration were calculated. Electronic patient files were used to obtain patient information, e.g., baseline characteristics or laboratory parameters.

Typically, the area under the curve (AUC)/MIC ratio is calculated to predict exposure of isavuconazole. Based on our data with only one plasma and ascites level of the antifungal, we calculated instead the Cmax/MIC ratio that expresses effectiveness of isavuconazole.

Although, data with plasma and ascites levels are from different timepoints, we calculated the Cmax/MIC in two affected patients with candida peritonitis to express the effectiveness of isavuconazole. In all other cases, only the penetration ratios were calculated (ascites drug level/plasma drug level). Variation of timepoint of paracentesis and sampling is explained by the circumstance that paracentesis was performed irrespective of the study based on the decision made by the treating ICU physician, e.g., of search for specific sepsis focus or other reasons, e.g., intra-abdominal fluid removal during mechanical ventilation problems.

We used IBM SPSS Statistics 25 (SPSS Inc., Chicago, IL, USA) for all statistical analyses in this study. Descriptive data of normally distributed parameters are presented as mean ± standard deviation and as median and range for non-parametric parameters.

## 3. Results

Paracentesis was performed with a median of 5.5 days (IQR 3.5–10) after starting of isavuconazole treatment in sixteen critically ill patients (see Table 1). Baseline characteristics, timepoint of isavuconazole treatment and of ascites removal, and cumulative dosage are summarized in Table 1. Isavuconazole plasma and ascites fluid levels, and penetration ratios obtained from paracentesis are listed in Table 2. In two out of sixteen patients, *Candida* spp., in detail *C. glabrata* and *C. tropicalis*, could be isolated (see Table 2). 

Isavuconazole levels in ascites fluid (0.36 to 2.71 µg/mL) were lower than simultaneous levels in plasma (0.48 to 11.49 µg/mL) (Table 2). The penetration ratio in the removed ascites fluid (mean 3.1 ± 0.7 L) was 36% with a high variation of 13 to 63% in all investigated patients. 

In the two patients with isolated *Candida* spp. from ascites fluid a Cmax/MIC Ratio in plasma of 560 for *C. glabrata* and 2166 for *C. tropicalis* could be observed. Ascites values and penetration ratio are presented in Table 2. The microbiological examinations of the ascites fluid of the other patients were all negative.

The initial elevated WBC cell count in ascites from 4.5 G/l (Patient 1) and 1.99 G/l (Patient 2) decreased 48 h later to 1.94 G/l (Patient 1) and 0.45 G/l (Patient 2), respectively.

## 4. Discussion

Understanding the tissue penetration of systemically administered antifungal agents is crucial for antifungal efficacy in patients with intraabdominal candida infections. 

Following the total Cmax/MIC ratios as presented above for our in-house MICs, the results are in line with isavuconazole levels of previous studies and comparable to EUCAST MIC’s [3,6]. Thus, the in vitro MICs exceed isavuconazole concentrations in plasma. However, the relevance of in vitro MICs for different tissues, e.g., ascites fluid are still unknown and at the moment not available [8]. 

Echinocandines are still the mainstay for the management of intra-abdominal *Candida* spp. infections, especially in critically ill patients [1,3,9].

Data on antifungal drug ascites fluid concentrations are limited and typically derived from peritoneal dialysis or surgical patients. [8].

Some data are available for Flucytosine and Fluconazole, which presented comparable plasma and peritoneal levels (85% in patients with Fluconazole) [10,11]. Intraperitoneal voriconazole levels reaching approximately 50% of plasma levels after oral administration [9]. The peritoneal concentrations of (liposomal) amphotericin B are variable, and have less than 50% of serum concentrations, and in some cases are undetectable [10,12]. 

The mean penetration ratio into ascites fluid in this study was 36% as compared to plasma isavuconazole concentrations with a high variation of 13–63%. 

Isavuconazole develops from the water soluble prodrug isavuconazonium and is highly protein bound (>99%), predominantly, to albumin, and has a large apparent volume of distribution (300–500 l) [13]. Thus, following the results from the study of Lee et al. with oral administrated radiolabeled isavuconazonium sulfate in rats, detectable radioactivity could be found within 1 h post dose in 56/65 examined tissues/fluids [6]. However, no data were available for ascites or peritoneal fluid [6]. 

The reason for different penetration rates might either be the fact that isavuconazole is not only bound to albumin, but also to other serum components, or a different binding potency of albumin in the presence of serum that might by influenced by critically ill circumstances, e.g., pH abnormalities and also based on different albumin levels in ascites fluid.

Although, we see the strength of our results our study has several limitations: First, the study is conducted as a single center study, second the study has an observational character without interventions and a small sample size and third variation of our measured isavuconazole levels may be based on the fact that timepoints of drug monitoring were not standardized in this study. 

## 5. Conclusions

In summary, the results of this study suggest that isavuconazole has a varied penetration into the ascitic fluid. However, overall success of treatment with isavuconazole in cases of Candida peritonitis depends on the interplay of susceptibility of the isolated species, the immune status of the host, and possibly other inter-related factors, which have to be investigated in further clinical studies.

## Figures and Tables

**Table 1:** Baseline characteristics, timepoint of isavuconazole treatment, timepoint of ascites removal and cumulative dosage, patients with *Candida* spp. peritonitis are underlined in red.

Patient	Sex	Age	Main Diagnosis	Underlying Immuno-Suppression	Day(s) of Isavuconazole Treatment	Cumulative Dose (mg)	Day(s) of Paracentesis after Starting Isavuconazole Treatment	Time from Isavuconazole Infusion (h)
1	M	37	NHL	N; CTx	14	3600	5	8
2	W	77	AML	N; allo-STx	17	4200	3	4
3	M	54	MDS	N; CTx	21	5000	11	14
4	M	77	NHL	N; CTx	7	2200	1	9
5	M	53	MDS	N; CTx	23	5400	14	2
6	M	70	ALL	N; allo-STx	29	6600	8	9
7	M	52	AML	N; allo-STx	30	6800	15	4
8	W	21	NHL	N; auto-STx	18	4400	2	6
9	M	62	ALL	N; CTx	21	5000	7	17
10	M	75	MDS	N; CTx	12	3200	5	8
11	M	54	AML	N; allo-STx	18	4400	3	2
12	M	70	MDS	N; CTX	28	6400	4	4
13	W	64	AML	N; CTx	14	3600	9	12
14	W	68	NHL	N; auto-STx	13	3400	6	8
15	M	71	MDS	N; CTx	18	4400	14	16
16	W	56	NHL	N; CTx	15	3800	4	3

NHL = non hodgkin lymphoma; AML = acute myeloid leukaemia; MDS = myelodysplastic syndrome; ALL = acute lymphoid leukaemia; allo-Stx = allogenic stem cell transplantation; auto-STx = autologous stem cell transplantation; N = neutropenia; CTx = chemotherapy. Patients with IAC underlined in red.

**Table 2:** Isavuconazole concentrations in plasma and ascites fluid, penetration rates and Cmax/MIC ratios.

	All Patients (*n* = 16)	Patient 1 (*C. glabrata* MIC 0.005)	Patient 2 (*C. tropicalis* MIC 0.0012)
**Isavuconazole** **Concentration:** **(mean, range)**			
**Plasma** **(µg/mL)**	3.08 ± 2.56	2.8	2.6
	(0.48–11.49)		
**Ascites fluid** **(µg/mL)**	1.06 ± 0.65	0.75	1.4
	(0.36–2.71)		
**Penetration ratio** **(Ascites drug level/Plasma drug level) (%)**	0.36 ± 0.15	0.26	0.53
	(0.13–0.63)		
**Cmax/MIC Ratio Plasma** Guideline * **(2µg/mL)**		400	1666
**Cmax/MIC Ratio** Plasma * **In house**		560	2166

* Following the total Cmax/MIC ratios as presented above for our in-house MICs, the results are in line with guideline (Cmax/MIC ratio calculated with isavuconazole levels of previous studies) and comparable to EUCAST MIC’s [3,6].

## Data Availability

All relevant data are made available in the manuscript.
